# Partial Substitution of Corn Grain in the Diet with Beet Pulp Reveals Increased Ruminal Acetate Proportion and Circulating Insulin Levels in Korean Cattle Steers

**DOI:** 10.3390/ani12111419

**Published:** 2022-05-31

**Authors:** Inhyuk Jeong, Sang Weon Na, Hyeok Joong Kang, Seung Ju Park, Da Jin Sol Jung, Seok Hyeon Beak, Jaesung Lee, Do-Hyun Kim, Hyun Jin Kim, Mohammad Malekkhahi, Kamburawala Kankanamge Tharindu Namal Ranaweera, Myunggi Baik

**Affiliations:** 1 Department of Agricultural Biotechnology and Research Institute of Agriculture and Life Sciences, College of Agriculture and Life Sciences, Seoul National University, Seoul 08826, Korea; dlsgurdldy@snu.ac.kr (I.J.); kerberosia@snu.ac.kr (S.W.N.); azuresz@naver.com (H.J.K.); cmhjpsj@snu.ac.kr (S.J.P.); jdjs777@snu.ac.kr (D.J.S.J.); buta100@snu.ac.kr (S.H.B.); wotjd8168@snu.ac.kr (J.L.); dohyuni91@snu.ac.kr (D.-H.K.); hyunjin2673@hanmail.net (H.J.K.); mmalekkhahi@gmail.com (M.M.); namal@uwu.ac.lk (K.K.T.N.R.); 2 Institute of Green Bio Science and Technology, Seoul National University, Pyeongchang-gun 25354, Korea

**Keywords:** acetate, byproduct, beef cattle, lipogenic potential, microbial population, rumen fermentation

## Abstract

**Simple Summary:**

Intramuscular fat content is one of the important beef quality traits due to its contribution to beef palatability, including flavor, tenderness, and juiciness. Intramuscular fat content is determined by fat synthesis using substrates such as acetate and glucose in ruminants. Beet pulp is a byproduct of sugar beet processing and contains abundant neutral detergent fiber and pectin, with these two components readily fermentable in the rumen. Beet pulp may be a useful energy source in cattle to produce acetate during ruminal fermentation, thereby aiding in lipogenesis (fatty acid synthesis). In this study, partial substitution of corn grain in the diet with beet pulp increased ruminal acetate proportion and circulating insulin levels in beef cattle. Beet pulp could be used as a lipogenic energy source without affecting growth performance in cattle.

**Abstract:**

We investigated the effects of the partial substitution of corn grain in the diet with beet pulp on growth performance, ruminal fermentation characteristics, microbial profiles, and blood lipogenic parameters in fattening steers. Twelve Korean cattle steers (body weight, 485 ± 19.32 kg; age, 18.0 ± 0.17 months) were equally divided into corn grain (CG) and beet pulp (BP) groups. Approximately 75% of dry matter of the requirement was offered as a concentrate portion, and the remaining 25% was offered as oat straw. Eighty percent of the concentrate portion was provided by a pelleted basal concentrate, and the remaining 20% with corn grain for the CG group, or 18% beet pulp plus 2.0% rumen-protected fat for the BP group, respectively, by top dressing. The experiment was conducted for 14 weeks, including a 2-week acclimation period. Growth rate was not affected by beet pulp feeding (*p* = 0.55). The molar proportions of ruminal acetate (*p* < 0.05) on wk 4, the relative abundances of ruminal cellulolytic bacteria, including *Fibrobacter succinogenes* (*p* = 0.01) and *Ruminococcus albus* (*p* = 0.04) on wk 12, and serum insulin concentrations (*p* < 0.05) on wk 12 were higher in the BP group than in the CG group, whereas the molar proportions of propionate (*p* < 0.05) on wks 8 and 12 and serum nonesterified fatty acids (*p* < 0.05) on wk 12 were lower in the BP group. Beet pulp could be used as a lipogenic energy source without affecting growth performance during the fattening period of cattle.

## 1. Introduction

Intramuscular fat (IMF) content is one of the important beef quality traits worldwide [1,2]. IMF content is closely related to beef palatability, including flavor, tenderness, and juiciness [1,3]. Lipogenesis in the animal body affects IMF content. Lipogenesis in ruminants generally occurs in adipose tissue via the conversion of acetate and glucose to fatty acids [4,5]. Glucose has been suggested to be the preferred precursor for synthesizing IMF in ruminants [4,6]. However, Nayananjalie [7] observed that acetate was more effective than glucose as the primary substrate for lipid synthesis in several fat depots, including intramuscular, subcutaneous, and visceral fat of Angus × Simmental steers. In addition, several studies reported that there were no differences in fat synthesis according to the type of precursor, which is acetate or glucose, depending on the depot in Korean cattle steers [8,9].

In cattle fed corn grain, a relatively high proportion of propionate is produced via ruminal fermentation, and corn grain has been used to improve IMF deposition in beef cattle [1,10]. Beet pulp is another potential concentrate with an energy value of approximately 85% of corn [11]. Dried beet pulp is a byproduct of sugar beet processing and contains up to 40% neutral detergent fiber and approximately 23% pectin on a dry matter (DM) basis; these two components are readily fermentable in the rumen [12]. In ruminants, the consumption of pectic substances led to the production of more acetate and less propionate compared with starch [13]. Therefore, beet pulp may be a useful energy source in cattle to produce acetate during ruminal fermentation [14]. Replacing grains with beet pulp is an alternative method to decrease feed costs while maintaining animal performance because the demand and cost of cereal grains, such as corn, have increased in the last decade [15]. Beet pulp can replace corn as an energy source in dairy cattle [14,16]. However, limited information on using beet pulp as an energy source in Korean cattle is available. Furthermore, little information is available on the effects of beet pulp on rumen microbial profiles and lipogenic parameters in beef cattle.

We hypothesized that feeding beet pulp instead of corn grain might change rumen fermentation parameters without affecting growth performance in beef cattle. It may increase ruminal acetate production and alter the lipogenic potential of beef cattle. Therefore, the objective of this study was to evaluate the effects of the partial substitution of corn grain with beet pulp on growth performance, ruminal fermentation, microbial characteristics, and blood lipogenic parameters in fattening Korean steers.

## 2. Materials and Methods

This experiment was approved by the Seoul National University Institutional Animal Care and Use Committee (SNU-180717-3), Republic of Korea, and was conducted according to the Animal Experimental Guidelines provided by the Seoul National University Institutional Animal Care and Use Committee.

### 2.1. Animals, Experimental Design, and Diets

The experiments were conducted at the Seoul National University Animal Farm of the College of Agriculture and Life Sciences, Pyeongchang, South Korea. A total of 12 Korean cattle (Hanwoo) steers with an average initial weight of 485 ± 19.3 kg and a mean age of 18.0 ± 0.17 months were used for the experiment. Steers were housed in a sawdust-bedded pen (5 × 20 m) indoors, under a roof, and with doors installed on both sides of the barn. During the 2-week adaptation period, approximately 75% of the DM requirement was provided as a concentrate portion (~1.3% of the bodyweight/animal/day), with the remaining portion (~25%) comprising oat straw (2 kg/animal/day). Of the concentrate portion, 80% was provided as a pelleted basal concentrate using an automatic feeding station (DeLaval ALPRO system; DeLaval, Tumba, Sweden), and the remaining 20% was in the form of corn grain by top dressing on roughage. After the pretreatment period, the steers (*n* = 6/group) were divided into a corn grain (CG) group and a beet pulp (BP) group. During the 12-week experimental period, both groups were fed the same basal concentrate to meet the 80% DM requirement of the concentrate portion (approximately 1.3% of bodyweight per animal) using an automatic feeding station. The CG group was supplemented with flaked corn for the remaining 20% DM requirement of the concentrate portion by top dressing on roughage, and the BP group was supplemented with 18% beet pulp plus 2.0% rumen-protected fat on a DM basis to provide a similar energy level as the CG group. The basal concentrate pellet contained 63.7 g/kg of ground corn and 294 g/kg of flaked corn (Table 1). Thus, about one-third (35.8%) of the total corn in the CG diet was substituted with beet pulp plus protected fat. The amount of concentrate portion (basal concentrate plus supplement) was adjusted to approximately 1.3% of the bodyweight of each animal at 4-week intervals. Meanwhile, a fixed amount of roughage (2.0 kg of oat straw/animal) was offered to both groups throughout the experimental period.

The chemical composition of the protected fat (DM, 99.0%; ether extract, 82.3%; rice hulls, 11%; calcium, 6.70%) was supplied by Eunjin International Bio Technology (ENERFAT, Eunjin Bio Co., Ltd., Cheonan-si, South Korea). The chemical composition of the feeds is listed in Table 1. Water was supplied freely via automatic drinkers. Equal amounts of oat straw (2 kg/day) and supplement (corn grain or beet pulp plus protected fat) were individually supplied twice daily, at 0800 h and 1600 h, in a separate bucket. We turned off the automatic feeding machines supplying the concentrate at 9 h and 4 h before the morning and afternoon feeding of the supplement and roughage, respectively, to stimulate supplement intake. The feed samples were collected monthly and stored at −20 °C until analysis. No roughage residual was found, and little dust was present in the oat straw. Thus, we ignored the dust portion of the roughage when measuring residual levels. Most of the animals consumed all the supplement (corn flakes or beet pulp plus fat) provided, but any refusals were collected every morning before feeding and weighed to measure the supplement intake. Individual daily intake of roughage and the supplement was recorded. Individual daily intake of the concentrate was controlled by individual neck sensors, setting the amount of concentrate in the automatic feeding system daily, and automatically recording using the online ALPRO system (DeLaval, Tumba, Sweden). Bodyweight was recorded before the morning feeding on the starting date of the experiment and on weeks 4, 8, and 12 thereafter. The average daily gain (ADG) was calculated as bodyweight gain divided by the number of experimental days (from the first to the last day of the feeding trial). Feed efficiency was computed as the ADG (kg) divided by the total DM intake (kg).

### 2.2. Blood Collection and Analyses

Blood samples were collected on the initial starting day and on weeks 4, 8, and 12 at around 8:00 am after 9 h fasting via jugular venipuncture into a nonheparinized vacutainer (20 mL; Becton-Dickinson, Franklin Lakes, NJ, USA). We collected blood at 9 h fasting since the automatic feeding method of concentrate may cause variation in metabolic parameters during the feed permission period. The serum was prepared by centrifugation (1500× *g* at 4 °C for 15 min) and stored at −80 °C until analysis. Detail methods and reagents used for analyses of triglycerides, glucose, total cholesterol, and nonesterified fatty acid (NEFA) are described by Kang et al. [19]. The analytical methods were validated in our laboratory, as reported previously [19]. Serum leptin and insulin concentrations were quantified using enzyme-linked immunosorbent assays according to the manufacturer’s instructions. Leptin was analyzed using the Bovine Leptin Kit (MyBioSource, San Diego, CA, USA), and insulin was analyzed using the Mercodia Bovine Insulin Kit (Mercodia AB, Uppsala, Sweden). The intra and interassay coefficients of variation for the insulin kit were 3.83% and 7.10% based on bovine serum samples, and those of leptin were <10%.

### 2.3. Rumen Fluid Collection and Analysis

Rumen fluid was collected using the oral stomach tube method at 3 h after the morning feeding [20]. The first 100 mL of rumen fluid was discarded to avoid saliva contamination, and then 200 mL of rumen fluid was collected. The pH of the rumen fluid was measured directly with a pH meter (Ohaus Corp., Parsippany, NJ, USA). The ruminal fluid was strained through four layers of cheesecloth, and then 4 mL was preserved for the ruminal ammonia nitrogen analysis by mixing with 4 mL of 0.2 N HCL, while 1 mL of rumen fluid was added to 0.2 mL of 25% metaphosphoric acid for the volatile fatty acid analysis and stored at −20 °C until analysis. Ammonia nitrogen was analyzed using a modified colorimetric method [21]. The volatile fatty acid concentrations were determined using an Agilent Tech 7890A gas chromatograph (Hewlett Packard, Waldbronn, Germany) with a SUPELCOWAX 10 Capillary GC Column. This method is described in detail in Kang et al. [22]. For the microbial analysis, rumen fluid samples were immediately frozen in liquid nitrogen upon collection and stored at −70 °C until further use.

Frozen rumen fluid was thawed on ice and centrifuged at 13,000× *g* and 4 °C for 30 min. The supernatant was discarded, and the pellet was ground in a mortar as liquid nitrogen was poured into the mortar. The powder was then stored at −70 °C until analysis. The ruminal microbial population was determined with genomic DNA (gDNA) extraction and quantitative real-time polymerase chain reaction (qPCR). First, gDNA was extracted from pellets of the rumen contents using the DNeasy PowerSoil Kit (Qiagen, Valencia, CA, USA) following the manufacturer’s protocol. Then, qPCR was performed using the modified method of Denman and McSweeney [23] and the QunatiTect SYBR Green RT-PCR Master Kit (Qiagen, Hilden, Germany). Briefly, qPCR analyses were conducted in a 25 µL total reaction volume containing 10 µL gDNA (1–20 ng), 12.5 µL SYBR Green RT-PCR Master Mix, and 1.25 µL forward and reverse primers. The primer information and the gDNA concentrations used for each microorganism are provided in the Supplementary Data (Table S1). The qPCR conditions were initial denaturation at 95 °C for 10 min, 40 cycles of denaturation (95 °C for 15 s), annealing (60 °C for 1 min), extension (60 °C for 1 min), and final melting (65–95 °C ). Genomic DNA was serially diluted 10-fold (from 20,000 pg to 0.020 pg) for the primer efficiency test. The PCR reactions were carried out with each primer pair using the templates of serially diluted genomic DNA. PCR was performed under the conditions described above. Primer efficiency (E) and the relative abundance of the target microbe (R) were calculated according to Makkar and McSweeny [24]:

$$E=10^{\left(-1/\mathrm{slope}\right)}$$

$$R\;=\frac{{\left(E_{\mathrm{target}}\right)}^{\Delta \mathrm{Ct}\;\mathrm{target}\left(\mathrm{control}\;-\;\mathrm{treatment}\right)}}{{\left(E_{\mathrm{reference}}\right)}^{\Delta \mathrm{Ct}\;\mathrm{reference}\;\left(\mathrm{control}\;-\;\mathrm{treatment}\right)}}$$

Primer efficiency for all genes was 80–110%, which is acceptable for qPCR (Supplementary Materials, Table S1). The bacterial 16S rRNA gene was used as the reference gene.

### 2.4. Analysis of the Chemical Composition of the Feeds

The DM (method 930.15), crude protein (Kjeldahl N × 6.25, method 981.10), ether extract (method 920.39), ash (method 942.05), starch (method 948.02), calcium (method 927.02), phosphorus (method 965.17), and acid detergent fiber contents (method 973.18) of the concentrate and oat straw were analyzed by the method of the Association of Official Agricultural Chemists [25]. The neutral detergent fiber contents of the concentrate and oat straw were determined using the ANKOM200 Fiber Analyzer (Ankom Technology Corp., Macedon, NY, USA) according to the methods described by Mertens [26].

### 2.5. Statistical Analysis

The number of animals needed to detect a significant difference was calculated using G*Power software [27]. The power analysis indicated that at least six animals per group were needed to detect a significant difference with a power of 0.8 and a type III error rate of 0.05. The steers were randomly assigned to two diet groups: CG and BP groups. The data on ruminal fermentation and blood metabolites were subjected to repeated measures analysis of variance using the MIXED procedure (PROC MIXED) in SAS (ver. 9.4; SAS Institute Inc., Cary, NC, USA) to investigate changes over time (weeks). The model included treatment (diet), time (sampling date), and the treatment × time interaction as the fixed effects, with animal as the random effect within a diet group. Three variance–covariance structures (autoregressive type 1, compound symmetry, and Toeplitz) were tested, and the covariance structure that minimized the Schwarz’s Bayesian information criterion was chosen. Feed intake, growth performance, and microbial population were analyzed using the above model without the effect of time. When a dietary treatment was found to have a significant effect, pairwise differences between means were assessed using the PDIFF procedure. A *p*-value ≤ 0.05 was considered to indicate significance, and 0.05 < *p* ≤ 0.10 indicated a tendency.

## 3. Results

### 3.1. Growth Performance

Average daily gain and feed efficiency (gain/feed) were not different (*p* ≥ 0.44) between the CG and BP groups (Table 2). The partial substitution of corn grain with beet pulp did not affect (*p* ≥ 0.17) either DM intake/day or intake/BW of concentrate, forage, or supplement.

### 3.2. Ruminal Fermentation Characteristics and Microbial Population

The pH, ammonia nitrogen concentration, and total volatile fatty acid concentration in the ruminal fluid were not affected (*p* ≥ 0.42) by feeding beet pulp (Table 3). Treatment and time interaction effect was observed (*p* = 0.006) for ruminal acetate proportion. The acetate proportion was higher (*p* < 0.05) in the BP group than in the CG group on week 4 but not on weeks 8 or 12. For the ruminal propionate proportion, tendencies of diet and time interaction (*p* = 0.07) and diet (*p* = 0.06) effects were observed. The propionate proportion was lower (*p* < 0.05) in the BP group than in the CG group on weeks 8 and 12 but not on week 4. The tendency of diet and time interaction effect was observed (*p* = 0.07) for the ruminal butyrate proportion. The butyrate proportion was lower (*p* < 0.05) in the BP group than in the CG group on week 4 but not on weeks 8 or 12. Diet and time interaction (*p* = 0.005) and diet (*p* = 0.02) effects were observed for the acetate to propionate ratio in rumen fluid. The ratio was higher (*p* < 0.05) in the BP group than in the CG group on weeks 4, 8, and 12.

**Figure 1:**
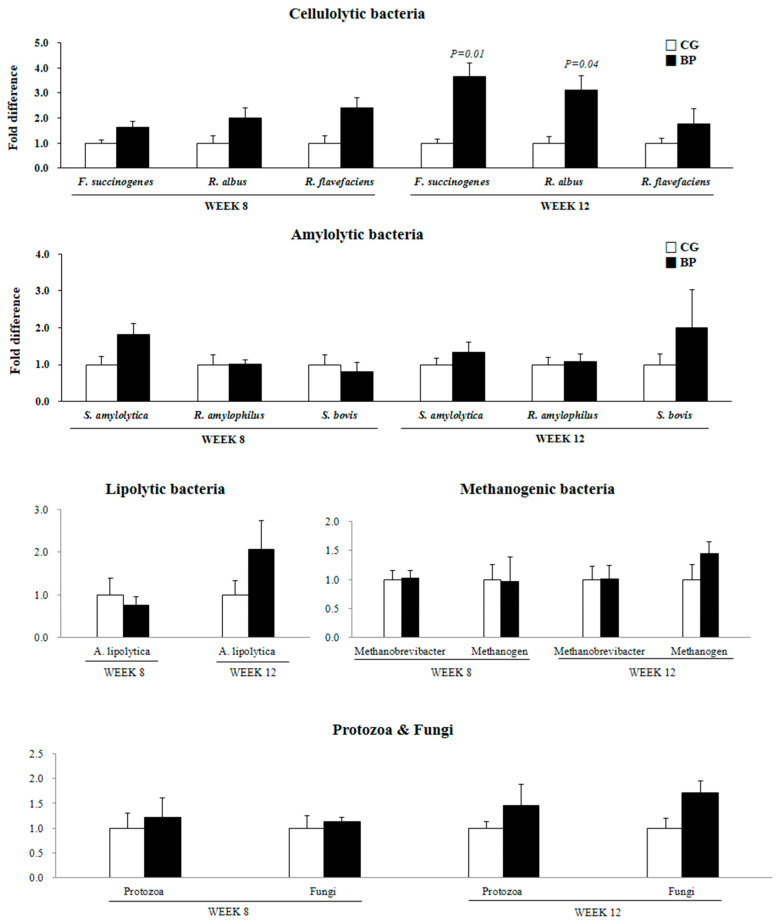
Effects of feeding corn grain (CG) or beet pulp (BP) on the ruminal population of cellulolytic bacteria, amylolytic bacteria, lipolytic bacteria, methanogens, total protozoa, and total fungi in Korean cattle steers. The microbial population was measured by quantitative real-time polymerase chain reaction using the total bacterial 16S rRNA gene as the reference gene. Fold difference of the CG group value of each bacterium for each week was normalized to 1.0-fold. Values are fold difference + standard error: *F. succinogenes* = *Fibrobacter succinogenes*; *R. albus* = *Ruminococcus albus*; *R. flavefaciens* = *Ruminococcus flavefaciens*; *S. amylolytica* = *Succinimonas amylolytica*; *R. amylophilus* = *Ruminobacter amylophilus*; *S. bovis* = *Streptococcus bovis*; *A. lipolytica* = *Anaerovibrio lipolytica*. Microbial population was not different (*p* > 0.05) if no *p*-value indicated.

The relative abundances of cellulolytic bacteria, including *Fibrobacter succinogenes* (*p* = 0.01) and *Ruminococcus albus* (*p* = 0.04), were higher in the BP group on week 12 compared with the CG group (Figure 1) but not on week 8, and no difference was observed for *Ruminococcus flavefaciens*. The relative abundances of amylolytic (*Succinimonas amylotica*, *Ruminobacter amylophilus*, and *Streptococcus bovis*) and lipolytic (*Anaerovibrio lipolytica*) bacteria, methanogens (methanogenic archaea and *Methanobrevibacter* spp.), fungi, and protozoa were not different (*p* > 0.10) between the two groups on weeks 8 and 12.

### 3.3. Blood Metabolites and Hormones

Treatment × time interaction was observed (*p* = 0.02) for serum cholesterol concentration (Table 4). The total serum cholesterol concentration was higher (*p* < 0.05) in the BP group than in the CG group on week 12 but not on weeks 4 or 8. Time (*p* = 0.004) and tendency of treatment (*p* = 0.10) effects were observed for serum NEFA concentration. The NEFA concentration was lower (*p* < 0.05) in the BP group than in the CG group on week 12 but not on weeks 4 or 8. Treatment (*p* = 0.03) and time (*p* = 0.001) effects were observed for serum insulin concentration. The insulin concentration was higher (*p* < 0.05) in the BP group than in the CG group on week 12 but not on weeks 4 or 8. The partial substitution of corn grain with beet pulp did not affect (*p* ≥ 0.11) glucose, triglyceride, or leptin concentrations at any of the time points.

## 4. Discussion

In this study, the partial substitution (about one-third) of total corn in the concentrate portion of the diet with beet pulp did not affect DM intake, ADG, or feed efficiency. Similarly, adding beet pulp did not affect feed intake in Egyptian buffalo calves [12] or Arabian male lambs [28]. Adding beet pulp at up to 32% of DM also resulted in no differences in ADG or feed efficiency in fattening bulls [29]. Collectively, beet pulp could be a good energy source for Korean cattle steers without affecting growth performance.

The major carbohydrates in beet pulp are pectin and hemicelluloses, which are less acidotic, and may have positive effects on ruminal pH [30,31]. Adding beet pulp at up to 100% of corn increased ruminal pH in Egyptian buffaloes [12]. However, in the present study, the partial substitution of corn with beet pulp did not affect ruminal pH in Korean cattle steers. Consistent with our study, replacing corn with up to 24% beet pulp did not affect the pH in dairy cows [32]. In our study, the pH range (6.43–6.99) in both groups was within normal conditions [18]. This result might be attributed to supplementation with sodium bicarbonate as a buffering material in our basal concentrate for maintaining a normal range of ruminal pH (6.5 to 7.0) [18].

In the present study, the substitution of corn grain with beet pulp resulted in an increased proportion of acetate in the rumen on week 4. By contrast, the proportion of propionate was lower in the BP group on weeks 8 and 12, and the proportion of butyrate was lower on week 4 compared with the CG group. In agreement with the current study, Voelker and Allen [32] reported that substituting pelleted beet pulp for high-moisture corn increased the molar proportion of acetate and decreased the proportion of propionate in the rumen. The greater proportion of acetate observed in the BP group was not surprising, as ruminal acetate is derived primarily from the fermentation of structural carbohydrates such as pectin [33]. Furthermore, higher fiber intake and lower non-fiber carbohydrate intake in the BP group relative to the CG group likely resulted in higher acetate levels and lower propionate and butyrate levels, as demonstrated by Alamouti et al. [34]. In the present study, the C2:C3 ratio was higher in the BP group than in the CG group on weeks 4, 8, and 12. Similarly, feeding beet pulp (110, 220, and 330 g) as a substitute for barley in the diet increased the ruminal C2:C3 ratio in Holstein steers [35]. In our study, a higher C2:C3 ratio in the BP group could be explained by the fact that beet pulp contains higher levels of neutral detergent fiber and lower levels of non-fiber carbohydrates or starch; thus, its consumption results in the production of more acetate and less propionate compared with the consumption of corn grain [34]. 

The populations of cellulolytic bacteria *F*. *succinogenes* and *R*. *albus* were higher in the BP group than in the CG group on week 12. These typical cellulolytic bacteria have genes encoding enzymes that degrade plant fiber [36,37]. Voelker and Allen [14] speculated that adding dietary beet pulp would increase the population of fibrolytic bacteria and fibrolytic enzyme activity by providing excess available substrate for fiber degraders. In the present study, the high fiber content in the BP group might have contributed to a higher population of these cellulolytic bacteria compared with the CG group. Therefore, the higher proportion of acetate in the BP group compared with the CG group could be explained by the higher population of cellulolytic bacteria.

The insulin concentration was higher in the BP group than in the CG group, whereas the NEFA concentration was lower in the BP group. Increased levels of insulin cause a decrease in circulating NEFA levels [38]. Insulin increases lipogenesis and inhibits lipolysis [39], and insulin has been suggested as an IMF deposition marker in cattle [40,41]. The higher insulin concentration and lower NEFA concentration in the BP group suggest that partial substitution of corn grain with beet pulp may improve lipogenic potential in Korean cattle. Further study is needed to determine the effects of feeding beet pulp on beef quality. The higher serum cholesterol in the BP group on week 12 compared with the CG group might be explained by the higher intake of ether extract (0.27 kg/d vs. 0.32 kg/d; *p* = 0.02).

## 5. Conclusions

The present findings reveal that feeding beet pulp increased ruminal acetate proportion and circulating insulin levels without affecting growth performance in Korean cattle steer. These changes may be beneficial for the production of high marbled beef. Further research is warranted to analyze the effect of beet pulp feeding on meat quality (e.g., IMF deposition) of Korean cattle steers.

## Figures and Tables

**Table 1:** Ingredient and chemical compositions of diets and individual feeds.

	Dietary Treatment		Individual Feed			
Item	Corn Grain	Beet Pulp	Concentrate	Corn Grain	Beet Pulp	Oat Straw
Ingredients, g/kg dry matter (DM)			-	-	-	-
Concentrate ^1^	600	600				
Oat straw	250	250				
Supplemented corn grain	150	0	-	-	-	-
Supplemented beet pulp	0	135	-	-	-	-
Protected fat	0	15				
Total	1000	1000				
Chemical composition, g/kg						
DM	890	900	878	856	894	911
Crude protein (CP)	127	122	160	84.0	90.9	53.9
Ether extract (EE)	34	34	41.4	35.6	8.00	19.4
Ash	54	58	72.2	11.2	45.0	35.1
Neutral detergent fiber (NDF)	310	350	238	102	425	640
Acid detergent fiber	149	162	86.4	27.9	231	403
Non-fiber carbohydrates (NFC) ^2^	475	436	488	767	431	252
Starch	320	204	333	720	9.8	14
Calcium	91	100	13.9	0.20	8.50	2.10
Phosphorus	40	40	5.80	2.30	0.70	1.50
Total digestible nutrient (TDN) ^3^, %	71.8	69.4	72.7	85.0	69.1	55.9
Metabolizable energy (ME) ^4^, MJ/kg	10.6	10.5				

^1^ Ingredient of concentrate (g/kg): Steamed flaked corn 294, corn gluten feed 268, ground wheat 131, wheat flour 108, ground corn 63.7, limestone 30, molasses 28, rice bran 19.7, palm kernel meal 14, condensed molasses solubles 13.5, distiller’s dried grains with solubles 10, sodium bicarbonate 6.6, whole cottonseed 5.5, palm oil 2.2, mineral-vitamin premix 2.1, salt 2.1, ammonium chloride 1.6. Mineral and vitamin premix contained Vit. A, 2,650,000 IU; Vit. D3, 530,000 IU; Vit. E, 1050 IU; Niacin, 10,000 mg; Mn, 4400 mg; Zn, 4400 mg; Fe, 13,200 mg; Cu, 2200 mg; I, 440 mg; Co, 440 mg (Grobic-DC, Bayer Health Care, Leverkusen, Germany); ^2^ NFC (g/kg) = 1000 − (CP + EE + ash + NDF); ^3^ TDN (%) of concentrate and other individual feeds were obtained from Cargill Agri Purina, Inc. (South Korea) and NRC values of nutrient composition tables, respectively [17]; ^4^ ME (Mcal/kg) = 0.82 × digestible energy (DE) (Mcal/kg) [18]; 1 kg of TDN = 4.4 Mcal of DE [18].

**Table 2:** Effects of feeding beet pulp in replacement of corn grain on growth performance of Korean cattle steers.

Items	Treatment ^1^			
	CG	BP	SEM	*p*-Value
Initial body weight, kg	484	485	5.37	0.98
Final body weight, kg	558	564	5.70	0.90
Average daily gain, kg	0.89	0.93	0.040	0.55
Total dry matter (DM) intake ^2^, kg/d	7.80	7.47	0.207	0.45
Concentrate DM intake, kg/d	4.75	4.58	0.153	0.61
Supplement DM intake, kg/d	1.23	1.07	0.05	0.17
Forage DM intake, kg/d	1.82	1.82	0.001	0.33
Total feed intake ^2^, g/kg of body weight	15.01	14.39	0.39	0.28
Concentrate DM intake, g/kg of body weight	9.14	8.82	0.290	0.61
Supplement DM intake, g/kg of body weight	2.37	2.07	0.107	0.17
Forage DM intake, g/kg of body weight	3.50	3.50	0.002	0.67
Feed efficiency (gain/feed)	0.115	0.123	0.004	0.44

*n* = 6 per group; ^1^ treatment: CG = 20% corn grain for concentrate portion; BP = 18% beet pulp plus 2.0% protected fat for concentrate portion; ^2^ total intake = concentrate + supplement (corn grain or (beet pulp + rumen-protected fat)) + forage.

**Table 3:** Effects of feeding beet pulp in replacement of corn grain on ruminal fermentation characteristics of Korean cattle steers.

Items	Treatment ^1^				*p*-Value		
	CG	BP	Mean	SEM	Treatment	Time	Treatment × Time
pH							
Week 0	6.54	6.50	6.52 ^y^	0.06	0.53	0.001	0.78
Week 4	6.74	6.89	6.81 ^x^	0.11			
Week 8	6.43	6.57	6.49 ^y^	0.09			
Week 12	6.91	6.99	6.94 ^x^	0.09			
Ammonia nitrogen, mg/dL							
Week 0	23.4	25.8	24.6	2.40	0.89	0.86	0.25
Week 4	32.3	20.6	26.5	1.70			
Week 8	23.2	21.4	22.3	2.29			
Week 12	19.2	26.9	23.1	5.70			
Total volatile fatty acid, mM							
Week 0	112	111	111	6.60	0.83	0.28	0.64
Week 4	123	125	124	5.40			
Week 8	127	122	124	5.70			
Week 12	109	123	115	9.20			
Acetate, mol/100 mol							
Week 0	59.2	58.8	59.0 ^z^	0.53	0.11	0.001	0.006
Week 4	60.0 ^b^	63.4 ^a^	61.7 ^y^	0.62			
Week 8	62.8	64.2	63.5 ^x^	0.51			
Week 12	62.9	64.1	63.5 ^x^	0.51			
Propionate, mol/100 mol							
Week 0	17.5	17.9	17.7 ^y^	0.55	0.06	0.001	0.07
Week 4	17.9	16.2	17.1 ^y^	0.57			
Week 8	22.2 ^a^	19.7 ^b^	20.9 ^x^	0.55			
Week 12	21.8 ^a^	19.7 ^b^	20.8 ^x^	0.45			
Butyrate, mol/100 mol							
Week 0	16.3	15.6	15.9 ^x^	0.01	0.13	0.001	0.07
Week 4	16.2 ^a^	13.4 ^b^	14.8 ^y^	0.01			
Week 8	12.0	13.4	12.7 ^y^	0.01			
Week 12	12.3	13.4	12.9 ^y^	0.01			
Acetate to propionate ratio							
Week 0	3.40	3.32	3.36 ^y^	0.09	0.02	0.001	0.005
Week 4	3.37 ^a^	3.98 ^a^	3.67 ^x^	0.15			
Week 8	2.85 ^b^	3.28 ^a^	3.06 ^y^	0.09			
Week 12	2.89 ^b^	3.26 ^a^	3.08 ^y^	0.08			

*n* = 6 per group; ^1^ treatment: CG = 15% corn grain for concentrate portion; BP = 13.5% beet pulp plus 1.5% protected fat for concentrate portion; ^a, b^ mean values with different letters in the same row differ (*p* < 0.05); ^x, y, z^ mean values with different letters in the same column differ (*p* < 0.05).

**Table 4:** Effects of feeding beet pulp in replacement of corn grain on blood metabolites of Korean cattle steers.

Item	Treatment ^1^				*p*-Value		
	CG	BP	Mean	SEM	Treatment	Time	Treatment × Time
Glucose, mg/dL							
Week 0	80.8	79.2	80.0	2.36	0.99	0.09	0.76
Week 4	73.5	74.8	74.1	1.18			
Week 8	77.2	76.2	76.7	2.42			
Week 12	74.7	75.0	74.8	1.19			
Triglyceride, mg/dL							
Week 0	23.3	20.7	22.0 ^y^	1.29	0.11	0.04	0.88
Week 4	24.3	19.7	22.0 ^y^	1.71			
Week 8	23.0	20.3	21.7 ^y^	1.70			
Week 12	18.8	16.0	17.4 ^x^	1.00			
Total cholesterol, mg/dL							
Week 0	130	123	127 ^y^	6.2	0.44	0.001	0.02
Week 4	158	145	151 ^y^	10.9			
Week 8	175	209	192 ^x^	11.6			
Week 12	170 ^b^	202 ^a^	156 ^x^	9.6			
Nonesterified fatty acids, mg/dL							
Week 0	134	137	136 ^x^	9.1	0.10	0.004	0.15
Week 4	105	90.5	98.3 ^y^	5.9			
Week 8	130	108	120 ^x^	14.5			
Week 12	173 ^a^	119 ^b^	148 ^x^	11.1			
Mean	135 ^a^	114 ^b^					
Leptin, ng/mL							
Week 0	9.79	9.01	9.40 ^x^	0.450	0.49	0.0008	0.94
Week 4	7.20	7.19	7.17 ^y, z^	0.673			
Week 8	6.23	5.55	5.89 ^y^	0.458			
Week 12	8.65	8.64	8.65 ^x, z^	0.500			
Insulin, ng/mL							
Week 0	1.08	1.27	1.18 ^x^	0.142	0.03	0.001	0.27
Week 4	1.16	1.54	1.35 ^x^	0.136			
Week 8	0.55	0.84	0.70 ^y^	0.082			
Week 12	0.79 ^b^	1.52 ^a^	1.15 ^x^	0.152			
Mean	0.90 ^b^	1.29 ^a^					

*n* = 6 per group; ^1^ treatment: CG = 15% corn grain for concentrate portion; BP = 13.5% beet pulp plus 1.5% protected fat for concentrate portion; ^a, b^ mean values with different letters in the same row differ (*p* < 0.05); ^x, y, z^ mean values with different letters in the same column differ (*p* < 0.05).

## Data Availability

Data will be made available upon reasonable request.

## Supplementary Materials

The following supporting information can be downloaded at: https://www.mdpi.com/article/10.3390/ani12111419/s1, Table S1: Primers, gDNA concentrations, and primer efficiency for the quantification real-time polymerase chain reaction assay.
