# Supplementary material for: Partial Substitution of Corn Grain in the Diet with Beet Pulp Reveals Increased Ruminal Acetate Proportion and Circulating Insulin Levels in Korean Cattle Steers
Source: Animals (Basel). 2022 May 31;12(11):1419. doi: 10.3390/ani12111419 (PMC9179527; doi:10.3390/ani12111419)
Supplement: Supplementary file 1 [file animals-12-01419-s001.zip › animals-1708904-supplementary.pdf]

Table S1. Primers, gDNA concentrations, and primer efficiency for the quantification real-time polymerase chain reaction assay

Microbes	gDNA (ng/10 ul)	Forward (F)/ Reverse (R)	Primer concentration	Primer sequence	Reference	Primer efficiency (%)
Total bacteria	1	F	0.3 uM	CGGCAACGAGCGCAACCC	Denman and McSweeney, 2006, FEMS Microbiol. Ecol. 58:572.	108
		R	0.3 uM	CCATTGTAGCACGTGTGTAGCC		
Total protozoa	20	F	0.3 uM	GCTTTCGWTGGTAGTGTATT	Sylvester et al., 2004, J. Nutr. 134: 3378.	80.3
		R	0.3 uM	CTTGCCCTCYAATCGTWCT		
Total fungi	10	F	0.3 uM	GAGGAAGTAAAAGTCGTAACAAGGTTTC	Denman and McSweeney, 2006, FEMS Microbiol. Ecol. 58:572.	96.1
		R	0.3 uM	CAAATTCACAAAGGGTAGGATGATT		
Methanogenic archaea	5	F	0.5 uM	GAGGAAGGAGTGGACGACGGTA	Ohene-Adjei et al., 2007. Appl. Environ. Microbiol. 73:4609.	95.5
		R	0.5 uM	ACGGGCGGTGTGTGCAAG		
Anaerovibrio lipolytica	10	F	0.5 uM	TGGGTGTTAGAAATGGATTCTAGTG	Khafipour et al., 2009, Appl. Environ. Microbiol. 75:7115	109
		R	0.5 uM	GCACGTCATTCGGTATTAGCAT		
Fibrobacter succinogenes	5	F	0.5 uM	GGAGCGTAGGCGGAGATTCA	Denman and McSweeney, 2006, FEMS Microbiol. Ecol. 58:572.	98.5
		R	0.5 uM	GCCTGCCCCTGAACTATCCA		
Ruminobacter amylophilus	5	F	0.3 uM	CTGGGGAGCTGCCTGAAT	Stevenson and Weimer, 2007, Appl. Microbiol. Biotechnol. 75,165.	91.8
		R	0.3 uM	CATCTGAATGCGACTGGTTG		
Ruminococcus albus	10	F	0.5 uM	CCCTAAAAGCAGTCTTAGTTCG	Koike and Kobayashi, 2001, FEMS Microbiol. Lett. 204:361	98.0
		R	0.5 uM	CCTCCTTGCGGTTAGAACA		
Ruminococcus flavefaciens	5	F	0.5 uM	CGAACGGAGATAATTTGAGTTTACTTAGG	Denman and McSweeney, 2006, FEMS Microbiol. Ecol. 58:572.	92.4
		R	0.5 uM	CGGTCTCTGTATGTTATGAGGTATTACC		
Streptococcus bovis	20	F	0.5 uM	TTCCTAGAGATAGGAAGTTTCTTCGG	Sylvester et al., 2004, J. Nutr. 134:3378	112
		R	0.5 uM	ATGATGGCAACTAACAATAGGGGT		
Succinimonas amylolytica	5	F	0.5 uM	CGTTGGGCGGTCATTTGAAAC	Khafipour et al., 2009, Appl. Environ. Microbiol. 75:7115	85.8
		R	0.5 uM	CCTGAGCGTCAGTTACTATCCAGA		
Methanobrevibacter spp.	10	F	0.3 uM	CCTCCGCAATGTGAGAAATCGC	Ramírez-Restrepo et al., 2016, Anim. Feed Sci. Tech. 216: 58.	94.6
		R	0.3 uM	TCWCCAGCAATTCCCACAGTT		
